# Treatment Modalities for Dementia in Down’s Syndrome: A Literature Review

**DOI:** 10.7759/cureus.27881

**Published:** 2022-08-11

**Authors:** Smriti Lamsal Lamichhane, Vaiishnavi Ramesh, Collins O Opara, Farhana Yaqoob Khan, Gargi Kabiraj, Humaira Kauser, Jaimee J Palakeel, Mazin Ali, Phani Chaduvula, Sanika Chhabra, Lubna Mohammed

**Affiliations:** 1 Internal Medicine, California Institute of Behavioral Neurosciences & Psychology, Fairfield, USA; 2 Family Medicine, California Institute of Behavioral Neurosciences & Psychology, Fairfield, USA; 3 Radiation Medicine, California Institute of Behavioral Neurosciences & Psychology, Fairfield, USA; 4 Pathology, California Institute of Behavioral Neurosciences & Psychology, Fairfield, USA; 5 General Practice, California Institute of Behavioral Neurosciences & Psychology, Fairfield, USA; 6 College of Medicine, California Institute of Behavioral Neurosciences & Psychology, Fairfield, USA; 7 Neurology, California Institute of Behavioral Neurosciences & Psychology, Fairfield, USA

**Keywords:** down's syndrome, alzheimer's disease, dementia, an acetylcholinesterase inhibitor, donepezil, memantine, exercise therapy, congenital anomaly, mongolism, trisomy 21

## Abstract

Down's syndrome (DS) is the most well-known chromosomal abnormality characterized by an extra chromosome 21 and multiple systemic issues. The higher production of amyloid precursor protein (APP), the precursor peptide of beta-amyloid, predisposes persons with DS to early Alzheimer's disease (AD). The prevalence of dementia has increased as a function of the extended life expectancy of persons with DS. Because we know little about the treatment of dementia in persons with DS, this review focuses on the pathophysiology and management strategies to improve the overall quality of life.

## Introduction and background

Down's syndrome (DS), also called Trisomy 21, is the most common genetic disorder affecting around one in 800 to one in 1000 births globally [1]. This syndrome results from the inheritance of an extra chromosome 21 or a translocated portion of chromosome 21. This leads to intellectual disability and physical abnormalities commonly characterized by [2] distinct facial features and stunted growth [3]. The syndrome may also be associated with congenital cataracts, heart defects, Hirschsprung’s disease, West syndrome, seizures, leukemia, sleep apnea, sensory deficiencies, autoimmune and endocrine pathologies, earlier aging, and cognitive impairment, and Alzheimer-type neuropathology [4]. With an improved life expectancy for people with DS, the risk of developing dementia at an earlier age is high. The prevalence is 10% between the ages of 40 to 49 years, 40% between the ages of 50-59 years, and 56% in those over 60 years [1,2].

People with DS may develop symptoms of Alzheimer's disease (AD) earlier in life than others because of the increased production of the amyloid precursor protein (APP), the precursor peptide of beta-amyloid located on chromosome 21. Increased protein expression of genes on chromosome 21 causes a cascade of effects on the fetal brain's structural development that impacts behavior throughout the lives of people with DS [4]. A recent study showed that increased levels of amyloid beta-peptide cause elevated levels of oxidative stress and subsequent neurodegeneration in older DS patients [5]. In addition to this genetic predisposition, studies proposed modifiable factors that also affect cognitive health and an increased risk of dementia, especially in sporadic onset AD. These modifiable risk factors include obesity, poor diet, smoking, alcohol consumption, cardiovascular disease, high blood pressure, and low level of socialization [1].

Physical activity is also an important risk factor that may have neuroprotective effects. Some studies suggest an inverse link between physical activity and dementia risk in the general population [1]. Although psychosocial treatments have been proven effective in helping patients manage their symptoms, dementia has no known cure. Cholinesterase inhibitors (donepezil, galantamine, and rivastigmine) and the N-methyl-D aspartate (NMDA) receptor antagonist, memantine, treat AD. Other therapies, including vitamin E, B, and statins, have yielded mixed results in clinical trials. It is also unclear whether any interventions could slow the progression of cognitive deterioration in people who have not been diagnosed with dementia [2,4,6]. This review aims to summarize the neuropathology, strategies to improve cognitive performance, and treatment modalities for dementia in DS.

Search methods

We collected and reviewed articles from 2009 to 2022. The database used was PubMed, Google Scholar, PubMed Central, and Science Direct. Keywords used: Down's syndrome, trisomy 21, mongolism, congenital anomaly, exercise therapy, memantine, donepezil, an acetylcholinesterase inhibitor, dementia, Alzheimer's disease.

## Review

This section will cover the pathophysiology of AD in those with DS as well as treatment options, including pharmacological and non-pharmacological approaches.

Pathogenesis of AD in people with DS

**Figure 1:**
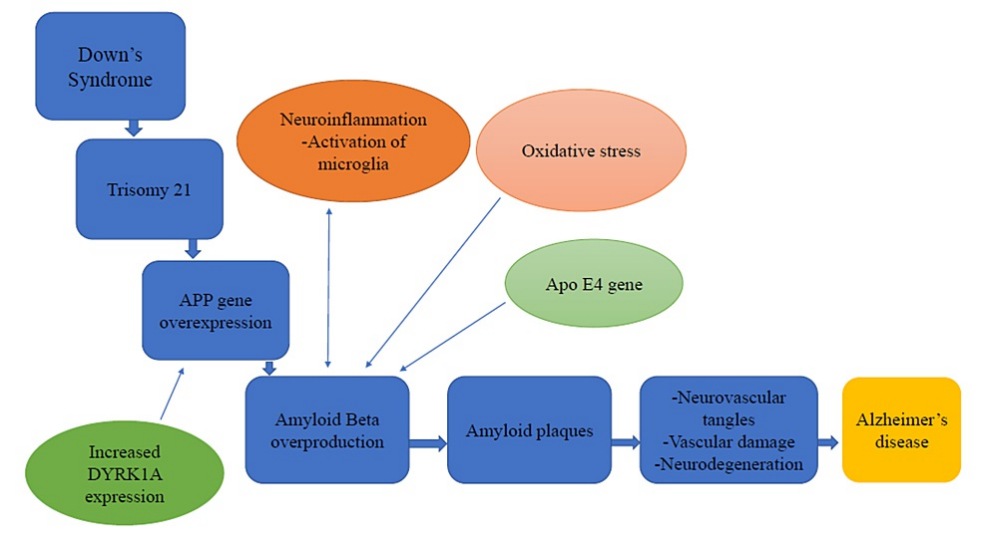
The Neuropathology of AD in people with DS. APP gene: Amyloid precursor protein gene, DYRK1A: Dual specificity tyrosine phosphorylation regulated kinase 1A, Apo E4: Apolipoprotein E4 The idea of the figure has been adopted from the review article [7].

AD developing in middle age is characterized by neuropathological changes in free radical metabolism, reduced mitochondrial activity, and neuronal degeneration. The molecular causes of AD are the subject of much investigation, and no one knows what causes the disease [5]. Figure 1 illustrates the neuropathology of AD in people with DS.

Overexpression of the APP gene appears to be a key component in the development of AD in DS [3]. Proteolytic enzymes alpha, beta, and gamma-secretase cleave the APP gene. Alpha-secretase and gamma-secretase are responsible for the normal cleavage of APP, which results in insoluble products. The APP gene is cleaved by Beta-secretase and Gamma secretase in the AD signaling pathway, resulting in insoluble monomers known as Beta-amyloid (Aβ) peptides. Because the gene for Beta secretase is on chromosome 21, the additional copy of the chromosome produces more Beta secretase, resulting in higher amounts of Aβ and subsequent amyloid plaques in DS [3]. Amyloid plaques develop neurofibrillary tangles between neurons, interrupting signaling between them [8] and triggering immunological responses that cause inflammation, vascular damage, and neuronal apoptosis [9]. The link between early-onset AD and mutations in the APP gene and presenilin genes was a focus of the amyloid cascade hypothesis. Given that the APP gene is located on chromosome 21 and, thus, is inherited in triplicate by people with DS together with other genes on chromosome 21, the higher risk of early-onset AD associated with DS highlights the APP gene as a critical factor [8,9].

Other variables, such as the apolipoprotein E (ApoE) genotype [10], play a role in the severity of AD pathology in both the general population and those with DS. ApoE is required for the preservation and regeneration of synaptic circuitry following damage. The ApoE gene produces three ApoE protein isoforms: ApoE-2, ApoE3, and ApoE4. Carriers of ApoE4 are at a higher risk of acquiring AD and coronary artery disease, but ApoE3 and ApoE2 give relative protection against both conditions [11]. The presence of ApoE4 carries an increased risk of AD, with a two-fold increase in the amount of amyloid deposited in the brain [9,11]. The presence of all four alleles is associated with an increased risk of AD in the general population and earlier onset in DS [1].

Neuroinflammation is also recognized to play a role in the etiology of AD, mainly through the activation of microglia [9]. Microglia are specialized brain cells that play a role in brain immune defense. Depending on the stimuli and context, it can participate in anti-inflammatory or pro-inflammatory signaling [12,13]. According to studies, an interaction between Aβ and microglia in AD exacerbates the inflammatory response. Microglia establish a barrier around the Aβ plaque to inhibit further spread while also attempting to clear the Aβ. Microglia can phagocytose Aβ after activation surrounding the Aβ plaque. Accumulation of Aβ in the microglia can lead to microglial cell death, increased inflammation, and the recruitment of more microglia, thereby perpetuating the inflammatory cascade. Additionally, activated microglia can initiate a pro-inflammatory response, releasing cytokines such as tumor necrosis factor-α (TNF-α) and interleukin 1β (IL-1β), as well as other substances, which cause tissue damage [13].

The oxidative stress theory may also explain the development of AD in DS patients. The imbalance between the creation of free radicals and antioxidant defense is known as oxidative stress (OS). Reactive oxygen species (ROS) like superoxide radicals, hydrogen peroxide (H2O2), and hydroxyl species, which induce neurodegeneration, are widely encountered during oxidative metabolism [5]. Antioxidant enzymes such as superoxide dismutase (SOD), glutathione peroxidase (GPx), and catalase (CAT) aid in the removal of ROS. Among them, the enzyme SOD is the most potential OS inducer. Three isoforms exist within the body: (1) intracellularly, copper/zinc SOD (also known as SOD1); (2) extracellularly, copper/zinc SOD; and (3) mitochondrially situated manganese SOD (also known as SOD2) [14]. According to research, SOD1 plays a vital role in accelerating the conversion of free radical superoxide to H2O2 in the cytosol. CAT and selenium-containing GPx convert H2O2 to water. The SOD 1 gene is found on chromosome 21 [15]. The tripling of chromosome 21, which contains the SOD-1 gene, causes an imbalance in the ratio of SOD-1 to CAT and GPx, which leads to H2O2 buildup [5]. The creation of large quantities of H2O2 results from increased SOD activity. Other enzymes, such as CAT, GPx, and thioredoxin peroxidase, can easily remove the enzyme. Increased H2O2 levels in DS are not balanced by increased CAT and GPx levels, leading to ROS overproduction [16].

Genetic factors are primarily responsible for the early onset of AD in DS. According to recent research, the dual-specificity tyrosine-phosphorylation regulated kinase 1A (DYRK1A) gene supplies instructions for nervous system development. In DS, it is expressed at a higher level. Its overexpression is linked to several cellular changes and cognitive impairments. Through tau hyperphosphorylation and amyloid pathogenesis, DYRK1A may play a substantial role in developmental brain abnormalities and early-onset neurodegeneration [4,17]. The fact that the number of DYRK1A-positive and 3-repeat (3R tau) positive neurofibrillary tangles are increased several-fold in DS implicates DYRK1A in the pathogenesis of AD.

Furthermore, overexpression of DYRK1A leads to increased phosphorylation of APP, which accelerates amyloidogenic amyloid precursor protein cleavage, raising Aβ40 and 42 levels and brain β-amyloidosis. Presenilin 1 (PS1), a critical trigger protein in the etiology of AD, is implicated in this process [4]. In addition, PS1 is a key component of the γ-secretase complex, which forms Aβ. Furthermore, the inhibition of DYRK1A improves cognitive behavior in various mice models of DS. Finally, a recent clinical experiment found that giving young individuals with DS epigallocatechin gallate (EGCG) (9 mg/kg/day), a DYRK1A inhibitor, enhanced visual recognition memory, working memory ability, and adaptive behavior [17].

The early onset of AD in DS is mainly due to genetic factors but also to nutritional and lifestyle factors: diets rich in fats and refined carbohydrates increase the accumulation of atheroma layers and hypercholesteremia; a deficiency of vitamins and minerals; and a lack of physical activities and exercises intended for normal mental development [15].

Management strategies

**Figure 2:**
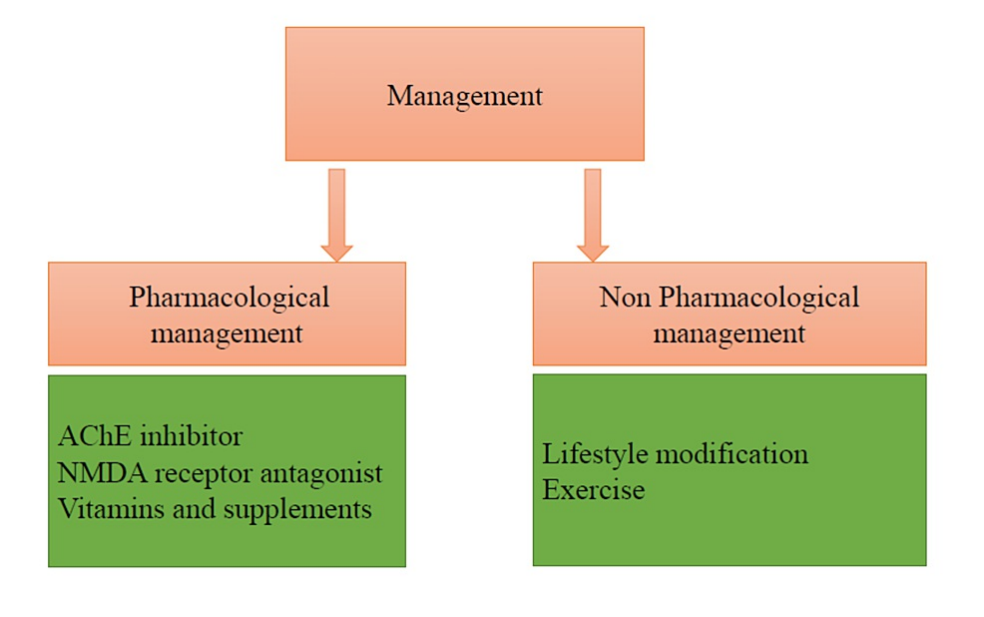
Management strategies AChE: Acetylcholinesterase, NMDA: N-methyl-D-aspartate Created by author Smriti Lamsal Lamichhane

AD is a progressive and irreversible disorder. Figure 2 illustrates the pharmacological and non-pharmacological approaches to the progression of dementia in AD.

Pharmacological management

Acetylcholinesterase (AChE) Inhibitors

AChE inhibitors, also called cholinesterase inhibitors, are a group of medicines that block the normal breakdown of the neurotransmitter acetylcholine, thereby increasing its synaptic concentration. Acetylcholine plays a vital role in learning, memory, and attention [18]. Although AChE inhibitors such as donepezil, galantamine, and rivastigmine are used as a treatment in Alzheimer's patients, their effects are under study in DS. Donepezil and rivastigmine are Food and Drug Administration (FDA) approved [10,19,20] and labeled for mild, moderate, and severe AD dementia. Galantamine, however, is approved only for mild and moderate AD [10].

Donepezil is a reversible and selective AChE inhibitor used to improve cognitive performance in mild to moderate types of AD. Therefore, people who do not have DS but have mild to severe AD may benefit from donepezil. On the other hand, people with DS tend to develop AD at a younger age than the general population and are physically different in size, metabolism, and heart rate; thus, they may have different needs [18]. A randomized controlled trial compared donepezil to a placebo group involving 30 persons with DS and AD. Sixteen people received donepezil, and 14 received a placebo. For the first four weeks of the trial, the dosage was 5 mg of donepezil per day, then 10 mg per day. The experiment lasted for 24 weeks. After a thorough search, they found that only one study was eligible for inclusion. There were no statistically significant results for any outcomes [18]. This review was superseded in 2015 by a new one [2]. The study compared the effectiveness of donepezil to the placebo group in four randomized controlled trials (192 participants). After 12-24 weeks, the results were summarized based on cognitive ability, behavioral difficulties, and adverse occurrences. There was no apparent benefit, but there was a risk of unpleasant outcomes [2]. Gastrointestinal disturbances, urine incontinence, muscle weakness [18], and a rise in heart vagal tone leading to bradycardia [2,12] are some of the side effects.

Galantamine is a selective, competitive, and rapidly reversible inhibitor of AChE that slows the breakdown of acetylcholine. Adverse effects include gastrointestinal disturbance [12].

Rivastigmine is a low-reversible carbamate inhibitor that inhibits both Butyrylcholinesterase and AChE, unlike donepezil, but its effects are shorter-lived [12,19]. It has the potential for transdermal absorption and is administered as a transdermal patch. It can be administered in individuals with renal or hepatic impairment, and it has no interactions with other drugs, making it potentially useful for patients with other co-morbidities [19]. A randomized controlled trial tested participants with AD in DS with rivastigmine. After comprehensive searches, however, there was no relevant study found for inclusion [19]. Although it is effective in treating AD, it has been demonstrated to hurt cognitive function [12]. In addition, gastrointestinal disruption, headache, dizziness [12], skin irritation, and dermatitis at the intradermal patch application site [10] are also some of the other side effects.

According to the recent Cochrane Reviews of donepezil and rivastigmine, there is insufficient data to make an educated decision about the potential utility of cholinesterase inhibitors for the treatment of dementia in adults with DS [2,18,19].

N-methyl-D-aspartate (NMDA) Receptor Antagonist

Memantine is an NMDA receptor antagonist that binds to NMDA receptors and stops glutamate from binding, preventing calcium from entering nerve cells. Two investigations in DS mice models have indicated that memantine improves cognitive function and neuropathology in AD [6]. Because preclinical research showed that memantine improved learning and memory in Ts65Dn mice, a randomized, double-blind, placebo-controlled trial was done on 40 people (range 18-30 years) to study improvement in hippocampal-dependent function. The California Verbal Learning Test-II (CTLV-II), a secondary memory outcome measure connected with the original hypothesis, showed a substantial effect of 16 weeks of memantine treatment [21] (Table 1). Another prospective randomized, double-blind, placebo-controlled trial in individuals with DS aged 40 and up found no benefit from memantine treatment [6] (Table 1). The trial involved 173 participants. Among them, 88 received memantine, and 85 received a placebo for 52 weeks. Computer-generated sequencing and a minimization algorithm were used to ensure a balanced allocation for five prognostic factors (sex, dementia, age group, total Down's syndrome attention, memory, and executive function scales [DAMES] score, and center.) [6]. Another study, a randomized, double-blind, placebo-controlled phase 2 trial, using either memantine (20 mg/day orally) or placebo for 16 weeks on adolescents and young adults (15-32 years) with DS, showed no cognition-enhancing effects [22] (Table 1). A Cochrane Review [2] cited the advantage of memantine in persons with moderate or severe AD who are already taking cholinesterase inhibitors. The role of memantine in treating cognitive loss in people with DS was not substantiated due to limited and inconclusive studies [23]. 

Vitamins and Supplements

Because patients with both DS and AD are very susceptible to OS, various studies were conducted on both groups to reduce oxidative damage and improve cognition [16]. In trials with severe and mild-to-moderate Alzheimer's dementia, high dose vitamin E (900 IU), an antioxidant, proved beneficial [2]. It is a powerful chain-breaking antioxidant that prevents the generation of ROS during fat oxidation and the spread of free radical reactions [16]. Vitamin E's tocopherol and tocotrienol isoforms have a variety of qualities, including significant antioxidant and anti-inflammatory properties, immunological modulation, cellular signaling, and cholesterol reduction. Vitamin E levels in the blood are lower in people with AD [24]. A study recently discovered vitamin E's role in mental illnesses underscoring its essential role in normal human brain functioning [16]. 

The homocysteine methionine cycle, which includes the production of S adenosylmethionine, a crucial substrate for deoxyribonucleic acid (DNA) methylation, requires the vitamin B group. Epigenetically, DNA methylation affects gene expression. Epidemiological studies have linked AD to less methylated DNA, which results in the overexpression of AD-related genes. Vitamin B6, B9, and B12 are particularly protective against AD [12]. In addition, high dosages of vitamin B12 and folate have been shown in a single study to slow the rate of brain shrinkage in mild cognitive impairment and provide cognitive benefits in those with high homocysteine levels [2]. However, contrary results derived from other studies show that the intake of only vitamin B is not very efficient in reducing dementia-like symptoms [12].

Low vitamin D levels are found in patients with AD and are linked to dementia and cognitive deficiency. Through binding to its own receptors, vitamin D has been implicated in amyloid plaque clearance, antiinflammation, oxidative stress reduction, and neuroprotection [12].

DYRK1A Inhibitor

DYRK1A plays a significant role in neurodegeneration. DYRK1A kinase activity is inhibited by the green tea natural compound EGCG [4,14,25]. EGCG is a more potent free radical scavenger than vitamin E or C [16]. A double-blind, randomized assignment of EGCG treatments against placebo showed immediate working memory performance improvement [4]. Several DS mouse models have demonstrated advances in DYRK1A research. Human studies, however, have been limited by the difficulty in obtaining cells from human brains. As such, only a few human cell models have been used to investigate the role of DYRK1A in DS [17]. When 10 micromolar EGCG was used against DYRK1A, the results demonstrated increased cell proliferation and decreased apoptosis. The positive results achieved with DS mouse models, and more recently, in human pluripotent stem cells from patients with DS (DS-iPSCs), give reason to believe that DYRK1A suppression could be a viable strategy for improving cognition in DS patients [17].

Coenzyme Q10

Coenzyme Q (CoQ) is a naturally occurring antioxidant in the human body. It combats OS in patients with DS by acting as a ROS scavenger. A study included children with DS to see how CoQ affected DNA damage as they got older. They received a daily dose of 4 mg/kg of CoQ or a placebo for six months. CoQ prevented oxidative damage to DNA pyrimidines in the younger age group (5-12 years) and reduced oxidized purines in the older age group (13-17 years) [14,16]. Another trial, including patients with DS aged 5 to 17 years old, was undertaken for 20 months with CoQ10 4mg/kg/day. They discovered some age-related variations in DNA oxidation [16]. Another study found that long-term therapy (i.e., four years) with CoQ10 at a dose of 4 mg/kg/day did no effect on DNA or ribonucleic acid (RNA) oxidation in children with DS [16,26].

Non-pharmacological management

There are numerous non-pharmacological methods for enhancing the quality of life for people who suffer from dementia. For example, adults with DS may address lifestyle changes, healthy eating habits, regular physical activity, and cognitive rehabilitation to improve cognitive abilities.

Lifestyle Modification

A healthy way of life is critical. Interventions that promote healthy and nutritious eating habits improve the overall health of AD patients [16]. Nutritional variables play a role at the beginning of AD in people with DS. For example, a diet high in lipids and straight-chain carbohydrates promotes atheromatous plaque formation and elevated cholesterol combined with vitamin and mineral deficits. Chromosome 21 dysfunction in DS subjects positively correlates with adverse lipid profiles like high triglyceride, low high-density lipoprotein levels, and elevated leptin levels [15]. To slow down AD in individuals with DS, parents should provide healthy food rich in vitamins, especially vitamin E (antioxidant), vitamin B group, minerals (especially magnesium), dietary fiber, and omega-3 fatty acids [16].

Exercise

Physical activity can be an effective non-pharmacological treatment for dementia prevention and management. According to a study, increased physical activity may be crucial for preserving cognitive components such as attention, memory, and executive function in people with AD [27,28]. Increased physical activity may also help prevent or delay AD onset by increasing hippocampal volume, improving cerebral perfusion, facilitating neurogenesis and synaptogenesis, and favorably reducing pathological changes such as Aβ accumulation [27]. Cohort research with persons with DS was done to examine the relationship between physical exercise and changes in dementia-related cognition and function [1]. Two hundred and fourteen adults with DS without dementia had their demographic, lifestyle, and health information collected at baseline followed by a two-year follow-up. The assessment involved using the Cambridge Examination for Mental Disorders of Older People with Down Syndrome and Others with Intellectual Disabilities (CAMDEX-DS) and genetic analysis. While adjusting for relevant variables, logistic regression models were used to investigate the potential correlations between the drop in CAMDEX-DS domains and exercise. At baseline, moderate-intensity exercise lowered the chance of everyday skill decline by 47%. In contrast, high-intensity exercise reduced the risk of personality and behavior drop to 62%. Following up, high levels of exercise lowered the chance of personality and behavior decline to 87%. At baseline, moderate-intensity exercise reduced the risk of memory and orientation deterioration to 62% during the follow-up period [1] (Table 1). A randomized trial to evaluate changes in cognitive function after completion of the 12-week exercise intervention was conducted remotely via video conferencing on a tablet computer [28] due to issues related to numerous barriers such as social support, the need for a caregiver, financial burden, and transportation. Cognitive function after 12 weeks was improved compared to baseline (Table 1) [28]. Another 12-month randomized trial [27] found that moderate to vigorous physical activity (MVPA) improved cognitive performance and quality of life in DS patients with AD (Table 1).

The summary of the intervention used to determine the effectiveness of management strategies and their outcomes is illustrated below in Table 1.

**Table 1:** Management strategies and their outcomes in Down’s syndrome people with dementia. AD: Alzheimer’s disease, DS: Down’s syndrome, MVPA: moderate to vigorous physical activity

Author	Year of Publication	Purpose of study	Intervention used	Conclusion
Costa A. C et al. [22]	2022	To discuss the effectiveness of memantine for dementia in 15-35 yrs participants with DS.	Memantine vs. placebo (Randomized, double-blind trial).	Cognitive enhancing effect not seen.
Pape SE et al. [1]	2021	To explore the association between regular exercise in DS people and changes in their dementia-related domains of cognition and function.	Cambridge Examination for Mental Disorders of Older people with DS and others with Intellectual Disabilities (CAMDEX-DS) and exercise.	A positive association between engaging in moderate and high levels of exercise and maintenance of memory, personality and behavior, and everyday skills in DS.
Ravancic M.E et al. [16]	2021	Importance of nutritional supplements for individuals with DS.	Vitamins as antioxidants.	There are possible benefits of some supplements, but there is not enough scientific evidence on them.
Ptomey L.T et al. [27]	2020	Increased MVPA to improve cognitive function and prevent AD in adults with DS.	Remote delivery of 12-month sessions to non-dementia adults and DS. Also, assess the impact of MVPA on cardiovascular fitness, quality of life, cognitive function, and brain parameters related to AD.	Increased MVPA may be effective in improving cognitive function, and quality of life on AD in DS.
Ptomey L T et al. [28]	2018	To evaluate changes in cognitive function after 12 weeks of exercise intervention delivered via video conferencing on a tablet computer in adults with DS.	Cognitive function was measured at baseline and end of the study using the Cantab Dementia Battery for iPads.	Increased physical activity may have positive changes in cognitive function.
Livingstone N et al. [2]	2015	To assess the effectiveness of donepezil for treating cognitive decline in people with DS.	Donepezil vs. placebo (Randomized Controlled Trial).	There was no apparent benefit, but there was a risk of unpleasant outcomes.
Hanney M et al. [6]	2012	To discuss the effectiveness of memantine for dementia in adults >40 years with DS.	Memantine vs. Placebo (Prospective, randomized double-blind trial).	Evidence is absent in cognitive improvement in DS patients older than 40 yrs.
Boada R et al. [21]	2012	To discuss the effectiveness of memantine for dementia in 40 young (range 18-30 years) people with DS.	Memantine vs. Placebo (Randomized double-blind trial).	The study determines a significant effect on one secondary memory outcome measure associated with the primary hypothesis, the California Verbal Learning test-II (CVLT-II).
Mohan M et al. [18]	2009	To determine the effectiveness and safety of donepezil for people with DS who develop AD.	Donepezil vs. placebo (Randomized Controlled Trial).	This study suggests possible benefits (but not statistically significant) in some individuals, but more extensive randomized controlled studies with longer-term follow-up are required.

Limitations

The main limitation of this review is that there is insufficient evidence on the role of pharmaceutical therapies in the management of dementia in people with DS. A considerable population with more power is required for research. This review article covers the positive association between exercise and improved cognitive function but does not provide much more clarity on the use of vitamins and supplements.

## Conclusions

This review focused on the pathophysiology and treatment options, including pharmacological and non-pharmacological options in reducing the progression of dementia in people with DS. Overexpression of APP appears to be a key component in developing Alzheimer's disease in people with DS. Within the context of the reported findings discussed above, pharmacological therapies such as Donepezil and Memantine may have a role in the management of dementia; however, research with more persons with DS is needed. Persons with DS are predisposed to oxidative stress and subsequent DNA damage. Vitamin E, DYRK1A inhibitor, and CoQ are antioxidants that help individuals with DS fight oxidative stress. There is a positive association between regular exercise and the maintenance of memory, personality, behavior, and conversational abilities in dementia-related cognition and function in adults with DS. Very little evidence exists for appropriate pharmacological treatment of cognitive impairment and dementia in people with Down's syndrome. Therefore, further research should focus on more extensive trials with well-powered cohorts to explore the prevention of cognitive decline in the DS population.

## References

[1] Pape SE, Baksh RA, Startin C, Hamburg S, Hithersay R, Strydom A (2021). The association between physical activity and CAMDEX-DS changes prior to the onset of Alzheimer’s disease in Down Syndrome. J Clin Med.

[2] Livingstone N, Hanratty J, McShane R, Macdonald G (2015). Pharmacological interventions for cognitive decline in people with Down syndrome. Cochrane Database Syst Rev.

[3] Malakooti N, Pritchard MA, Adlard PA, Finkelstein DI (2014). Role of metal ions in the cognitive decline of Down syndrome. Front Aging Neurosci.

[4] de la Torre R, Dierssen M (2012). Therapeutic approaches in the improvement of cognitive performance in Down syndrome: past, present, and future. Prog Brain Res.

[5] Perluigi M, Butterfield DA (2012). Oxidative stress and Down syndrome: a route toward Alzheimer-like dementia. Curr Gerontol Geriatr Res.

[6] Hanney M, Prasher V, Williams N (2012). Memantine for dementia in adults older than 40 years with Down's syndrome (MEADOWS): a randomized, double-blind, placebo-controlled trial. The. Lancet.

[7] Puttagunta SM, Islam R, Kundu S (2022). Tiny toes to tau tangles: Down's syndrome and its association with Alzheimer's disease. Cureus.

[8] Priebe GA, Kanzawa MM (2020). Reducing the progression of Alzheimer's disease in Down syndrome patients with micro-dose lithium. Med Hypotheses.

[9] Castro P, Zaman S, Holland A (2017). Alzheimer's disease in people with Down's syndrome: the prospects for and the challenges of developing preventative treatments. J Neurol.

[10] Atri A (2019). The Alzheimer’s disease clinical spectrum: diagnosis and management. Med Clin North Am.

[11] Jeremic D, Jiménez-Díaz L, Navarro-López JD (2021). Past, present and future of therapeutic strategies against amyloid-β peptides in Alzheimer's disease: a systematic review. Ageing Res Rev.

[12] Ghosh S, Durgvanshi S, Agarwal S, Raghunath M, Sinha JK (2020). Current status of drug targets and emerging therapeutic strategies in the management of Alzheimer's disease. Curr Neuropharmacol.

[13] Kloske CM, Wilcock DM (2020). The important interface between apolipoprotein E and neuroinflammation in Alzheimer’s disease. Front Immunol.

[14] Muchová J, Žitňanová I, Ďuračková Z (2014). Oxidative stress and Down syndrome. Do antioxidants play a role in therapy?. Physiol Res.

[15] Mazurek D, Wyka J (2015). Down syndrome-genetic and nutritional aspects of accompanying disorders. Roczniki Państwowego Zakładu Higieny.

[16] Ravancic ME, Obradovic V (2021). Usage of nutritional supplements for individuals with Down syndrome. Progress in Nutrition.

[17] Feki A, Hibaoui Y (2018). DYRK1A protein, a promising therapeutic target to improve cognitive deficits in Down syndrome. Brain Sci.

[18] Mohan M, Carpenter PK, Bennett C (2009). Donepezil for dementia in people with Down syndrome. Cochrane Database Syst Rev.

[19] Mohan M, Bennett C, Carpenter PK (2009). Rivastigmine for dementia in people with Down syndrome. Cochrane Database Syst Rev.

[20] Stephens MM, Herge E, Wright C (2021). Down syndrome and dementia: A patient and care-giver centered approach. Dela J Public Health.

[21] Boada R, Hutaff-Lee C, Schrader A, Weitzenkamp D, Benke TA, Goldson EJ, Costa AC (2012). Antagonism of NMDA receptors as a potential treatment for Down syndrome: a pilot randomized controlled trial. Transl Psychiatry.

[22] Costa AC, Brandão AC, Boada R (2022). Safety, efficacy, and tolerability of memantine for cognitive and adaptive outcome measures in adolescents and young adults with Down syndrome: a randomized, double-blind, placebo-controlled phase 2 trial. Lancet Neurol.

[23] Mohan M, Bennett C, Carpenter PK (2009). Memantine for dementia in people with Down syndrome. Cochrane Database Syst Rev.

[24] Browne D, McGuinness B, Woodside JV, McKay GJ (2019). Vitamin E and Alzheimer’s disease: what do we know so far?. Clin Interv Aging.

[25] Franceschi C, Garagnani P, Gensous N, Bacalini MG, Conte M, Salvioli S (2019). Accelerated bio-cognitive aging in Down syndrome: State of the art and possible deceleration strategies. Aging Cell.

[26] Larsen EL, Padella L, Bergholdt HK (2018). The effect of long-term treatment with coenzyme Q10 on nucleic acid modifications by oxidation in children with Down syndrome. Neurobiol Aging.

[27] Ptomey LT, Szabo-Reed AN, Martin LE (2020). The promotion of physical activity for the prevention of Alzheimer's disease in adults with Down Syndrome: Rationale and design for a 12 Month randomized trial. Contemp Clin Trials Commun.

[28] Ptomey LT, Szabo AN, Willis EA, Gorczyca AM, Greene JL, Danon JC, Donnelly JE (2018). Changes in cognitive function after a 12-week exercise intervention in adults with Down syndrome. Disabil Health J.

